# Interaction of miR-146a-5p with oxidative stress and inflammation in complications of type 2 diabetes mellitus in male rats: Anti-oxidant and anti-inflammatory protection strategies in type 2 diabetic retinopathy

**DOI:** 10.22038/IJBMS.2021.56958.12706

**Published:** 2021-08

**Authors:** Seyed Ahmad Rasoulinejad, Abolfazl Akbari, Khadijeh Nasiri

**Affiliations:** 1 Department of Ophthalmology, Ayatollah Rouhani Hospital, Babol University of Medical Sciences, Babol, Iran; 2 Department of Physiology, School of Veterinary Medicine, Shiraz University, Shiraz, Iran; 3 Department of Exercise Physiology, Faculty of Sport Sciences, University of Mazandaran, Babolsar, Iran

**Keywords:** Diabetic retinopathy, Inflammation, MicroRNA, Oxidative stress, Type 2 diabetes

## Abstract

**Objective(s):**

This study aimed to evaluate the role of miR-146a-5p in the pathogenesis of diabetic retinopathy and its interaction with oxidative stress and inflammation in the ocular tissue of rats with type 2 diabetes mellitus (T2DM).

**Materials and Methods:**

Twenty adult male Sprague Dawley rats (220 ±20 g) were randomly assigned to control and diabetic groups. A high-fat diet was used for three months to induce T2DM which was confirmed by the HOMA-IR index. After that, the levels of glucose and insulin in serum, HOMA-IR as an indicator of insulin resistance, the ocular level of oxidative markers, TNF-α, IL-1β, MIPs, and MCP-1 along with ocular gene expression of NF-κB, Nrf2, and miR-146a-5p were evaluated.

**Results:**

The level of lipid peroxidation along with metabolic and inflammatory factors significantly increased and the antioxidant enzyme activity significantly decreased in diabetic rats (*P*<0.05). The ocular expression of NF-κB and TNF-α increased and Nrf2, HO-1, and miR-146a-5p expression decreased in diabetic rats (*P*<0.05). In addition, a negative correlation between miR-146a-5p expression with NF-κB and HOMA-IR and a positive correlation between miR-146a-5p with Nrf2 were observed.

**Conclusion:**

It can be concluded that miR-146a-5p may regulate Nrf2 and NF-κB expression and inflammation and oxidative stress in the ocular tissue of diabetic rats.

## Introduction

Type 2 diabetes mellitus (T2DM) as a public health hazard is a chronic multifactorial disease whose occurrence depends on environmental and genetic factors. The number of diabetics is projected to reach 552 million by 2030 (1). Microvascular complications such as retinopathy, nephropathy, and neuropathy are mainly caused by diabetes-induced metabolic disorders and inflammation. Endothelial dysfunction (ED), cellular aging, and chronic and low-grade systemic inflammation are the main causes of vascular complications. However, targeting these pathophysiological pathways using new therapies has had little success in improving diabetes and its complications(2). This may be due to the involvement of other unknown mechanisms that contribute to the pathogenesis of this disease. In addition, even if combination therapy with glucose and lipid-lowering drugs and antihypertensive drugs can improve diabetes, vascular complications cannot be prevented, especially in patients with long-term disease (3). Diabetic retinopathy is one of the main microvascular complications of T2DM that causes blindness in middle-aged diabetic patients. Hyperglycemia, hypertension, ocular oxidative stress, and inflammation along with epigenetic mechanisms are the main causes of diabetic retinopathy. Chronic hyperglycemia, chronic low-grade inflammation, and oxidative stress through methylation of DNA and non-coding RNAs are the main epigenetic mechanisms of vascular complications of diabetes (4). Non-coding RNAs, including microRNAs (miRNAs), play a significant role in the pathogenesis of damage induced by hyperglycemia (5). A single miRNA can target multiple different cellular pathways and cellular responses to environmental stimuli by modulating promoter methylation, transcription, and processing (6). MiRNAs negatively regulate gene expression and provide highly efficient systemic communication in the body (3). Because of their easy detection, they are used as minimally invasive biomarkers to assess many diseases, including diabetes (3). Hyperglycemia, oxidative stress, and inflammation, which are known to cause diabetes complications, can affect the circulating miRNA profile. Therefore, in addition to their diagnostic value, they can be potential targets for controlling the complications of diabetes and metabolic diseases. It was reported that miR-125b, miR-146a-5p, and miR-29a-3p are involved in diabetic retinopathy (7). Some of these miRNAs regulate inflammatory pathways by the nuclear factor kappa B (NF-κB) (8). The activity of miR-146a-5p is related to the regulation of inflammation and senescence (9). In humans, miR-146a-5p is mainly expressed in endothelial cells and white blood cells and inhibits inflammation by down-regulating the expression of NF-κB, and attenuates the complications of diabetes (3, 7). It is well documented that the NF-κB/tumor necrosis factor (TNF)-α pathway is involved in the inflammatory status of diabetic patients. Oxidative stress is another mechanism that is also involved in the complications of diabetes. The major complications of oxidative stress-induced diabetes are regulated by the nuclear factor erythroid 2-related factor 2 (Nrf2)/Heme oxygenase-1 (HO-1) pathway. This pathway plays a key role in preventing diabetic vascular complications (10). These findings showed that a decrease in miR-146a-5p may play an important role in the onset and development of complications of diabetes. However, many ambiguities remain regarding functional impairment or its normal functions in type 2 diabetic retinopathy (10, 11). Therefore, this study aimed to evaluate the role of miR-146a-5p in the pathogenesis of diabetic retinopathy and its interaction with oxidative stress and inflammation pathways in the ocular tissue of rats with T2DM. 

## Materials and Methods

***Animal and study design***

All stages of this study were performed based on the guide for the care and use of laboratory animals approved by Shiraz University of Medical Sciences with reference number (SUMS. REC. 1398.07). Twenty adult male Sprague Dawley rats (220±20 g) were kept at 20 ± 2 °C and 12 hours light: 12 hours dark light cycle. Animals had free access to chow and water. After one week of familiarization, the animals were randomly divided into healthy control rats and type 2 diabetic rats.

***Induction of type 2 diabetes mellitus***

Induction of T2DM was performed by feeding a high-fat diet (HFD) for three months to normoglycemic adult male Sprague-Dawley rats (12, 13). To confirm the induction of T2DM, the levels of fasting glucose and insulin were measured and Homeostatic Model Assessment for Insulin Resistance (HOMA-IR) was then calculated (14). The serum level of fasting glucose and insulin were evaluated by a digital glucometer and a rat-specific kit (Shanghai Crystal Day Biotech Co., Shanghai, China), respectively. Food intake and body weight gain as another factor influencing insulin resistance and T2DM were weekly measured. Previous studies have shown that eating an HFD for three months or less can cause eye damage caused by diabetes (15, 16). Various models, including genetic models (16) and models induced by HFDs and streptozotocin injection (16, 17), have been reported in several studies. However, induction of eye damage, especially diabetic retinopathy, by HFDs is one of the most commonly used methods (12, 15, 16). In these studies, the use of high-fat food at different time ranges, including 2 months (15), 3 months (18), and even 7 months (12) has been used to induce retinopathy.

***Blood and tissue sampling***

After the last day of study and one night of fasting, the animals were anesthetized with a combination of ketamine (70 mg/kg) and xylazine (3-5 mg/kg) by intraperitoneal injection. Blood samples were collected directly from the ventricle with a syringe (5 ml) and poured into tubes. They were then centrifuged (3000 rpm, 10 min) and the sera were stored at -80 °C for later biochemical test. The left eye tissue was also removed quickly, washed in normal saline, and put in RNase-free and DNase-free microtubes, and was then stored quickly in liquid nitrogen.

***Biochemical assessment***

Serum levels of lipid profiles including total cholesterol (TC), triglyceride (TG), low-density lipoprotein (LDL), and high-density lipoprotein (HDL), and liver enzymes such as Alanine transaminase (ALT) and Aspartate transaminase (AST) were measured using colorimetric methods. The levels of antioxidant enzymes (superoxide dismutase (SOD), glutathione peroxidase (GPx) and catalase (CAT)), and malondialdehyde (MDA) were measured to assess ocular oxidative stress status. The activity of SOD and GPx were measured using a laboratory kits from Randox Company, UK. The method described by Aebi with little modification was used to measure the level of catalase (19). MDA content was measured by a spectrophotometric method described by Zal *et al*. (2007) (20). Total protein content in homogenized eye tissue was measured by Lowry *et al*. method (21). The levels of TNF-α, interleukin (IL)-1β, monocyte chemoattractant protein-1 (MCP-1), and macrophage inflammatory protein-1 (MIP-1)-α to evaluate ocular inflammation status were measured by enzyme-linked immunosorbent assay (ELISA) rat-specific kits (Shanghai Crystal Day Biotech Co., Shanghai, China). 

***Molecular evaluations***

Total RNA and miRNA were extracted using an RNA extraction kit (miRNeasy Mini Kit, Qiagen, Germany) from eye samples. RNA integrity and concentration were investigated using gel electrophoresis and a Nanodrop device (Nano Mabna Iranian, Iran), respectively. The oligonucleotide primers used in the real-time PCR reaction are presented in Table 1. The Real-time PCR reaction was performed by Rotor-Gene 6000 (Corbett Research, Australia). All samples were run in duplicate. For Quantitative Real-Time PCR, 50 ng cDNA and 0.5 μl of each primer (10 pmol) were used in a 20 μl reaction volume containing SYBR Green PCR Master Mix (BioFACT™ 2X Real-Time polymerase chain reaction (PCR) Master Mix, South Korea). To normalize the mRNA expression level of target genes, β-actin as a housekeeping gene was used. Relative expression was assessed by the comparative threshold cycle (Ct) and 2^−ΔΔCt^ method; the results displayed the fold change of gene expression. For microRNA analysis, the Mir-X miRNA First-Strand Synthesis Kit (TaKaRa, Japan) was used to synthesize the cDNA. The miR-146a-5p and U6 forward and reverse primers were used from the study of Wei *et al*., (22). Real-time PCR of miR-146a-5p was carried out with the Mir-X miRNA qRT-PCR SYBR Kit (TaKaRa, Japan). U6 RNA was used to normalize miR-146a-5p expression. Relative changes in expression of miR-146a-5p were assessed through the 2^−ΔΔCt^ method. 

***Histopathological examination***

The right eye was completely removed from the skull and fixed in a 10% formalin solution. It was then embedded in paraffin after a week, and sections with a thickness of 5 microns were prepared and then stained with hematoxylin and eosin. Histological features of the eye tissue in control and type 2 diabetic rats were examined with a light microscope (BX-51, Olympus Corporation, Tokyo, Japan) with the same magnification (40x). 

***Statistical analysis***

All data were recorded in SPSS software version 17 and analyzed using independent t-test and paired t-test. The correlations between miR-146a with HOMA-IR, NF-κB, and Nrf2 were calculated by Pearson correlation analysis. *P*<0.05 was considered as the minimum significant level. The results were expressed as mean ± standard deviation (SD).

## Results

***Evaluation of body weight, food intake, and metabolic factors in control and diabetic rats***

**Figure 1:**
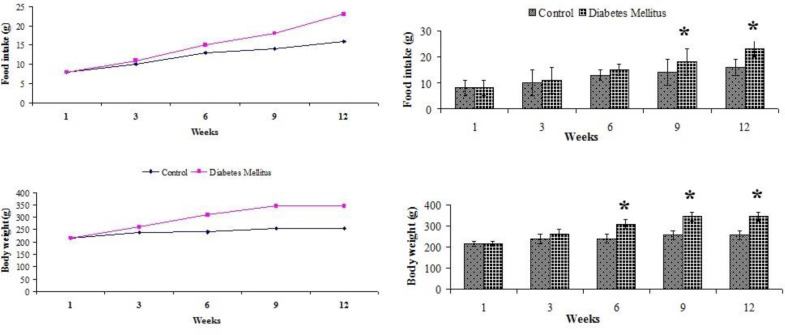
Mean ± SD of body weight (g) and food intake (g) in control and diabetic groups of rats

**Figure 2:**
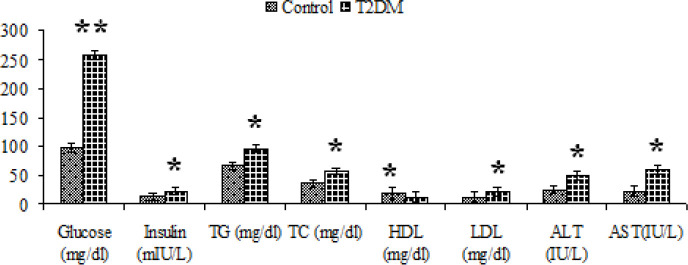
Mean ± SD of the serum levels of glucose, insulin, liver enzymes, and lipid profile in control and diabetic groups of rats

Food intake and weight gain significantly increased in the diabetic group compared with the control group (*P*<0.05, Figure 1). The levels of lipid profile, glucose, insulin, and liver enzymes were measured as metabolic factors to evaluate type 2 diabetes status. The mean ± SD of these variables in control and diabetic rats are presented in Table 2 and Figure 2. The levels of glucose, insulin, and HOMA-IR index, measured to evaluate insulin resistance, significantly increased in the diabetic group compared with baseline level and control group (*P*<0.05, Table 2 and Figure 2). The levels of TC, TG, and LDL significantly increased in the diabetic group compared with the control group, while HDL significantly decreased in the diabetic group compared with the control group (*P*<0.05, Figure 2). The serum levels of ALT and AST as indicators of liver damage significantly increased in the diabetic rats (*P*<0.05, Figure 2).

***Evaluation of oxidative damage and inflammation status in the ocular tissue of healthy and type 2 diabetic rats***

The mean± SD levels of SOD, GPx, CAT, and MDA are presented in Table 2. The activity of these enzymes significantly decreased in diabetic rats. MDA level significantly increased in the diabetic rats (*P*<0.05, Table 3). 

The mean± SD levels of TNF–α, IL-1β, MIP-1α, and MCP-1 as inflammatory biomarkers are presented in Table 4. The levels of these biomarkers significantly increased in the diabetic rats (*P*<0.05, Table 4).

***Expression of NF-*** ***κB*** ***, Nrf2, and miR-146a-5p was significantly altered in the ocular tissue of the diabetic group***

**Figure 3:**
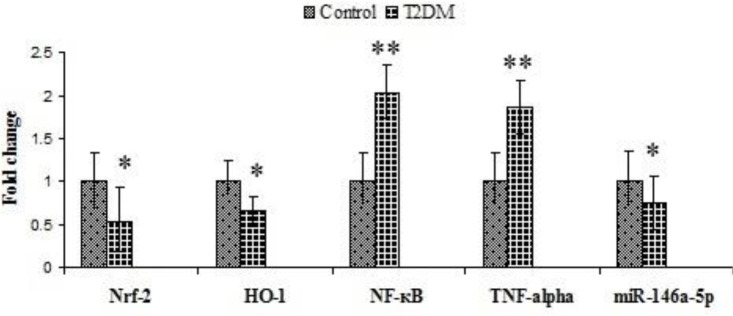
Mean ± SD of the gene expression of NF-κB, TNF-alpha, Nrf-2, HO-1, and miR-146a-5p in control and diabetic groups of rats

Expression of Nrf-2, HO-1, and miR-146a-5p significantly decreased in the ocular tissue of the diabetic rats, and conversely, expression of NF-κB and TNF-α significantly increased in this group (*P*<0.05, Figure 3).

***Investigating the correlation between miR-146a-5p expression and inflammation, oxidative stress, and insulin resistance***

In addition, Pearson correlation between miR-146a-5p with Nrf2, NF-kB and HOMA-IR was calculated. The results showed a negative correlation between miR-146a-5p with NF-κB (*r*: -0.53, *P*=0.032) and HOMA-IR (*r*: -0.58, *P*=0.038) and a positive correlation between miR-146a-5p with Nrf2(*r*: +0.51, *P*= 0.043).

***Histological features of the eye in type 2 diabetes retinopathy***

**Figure 4:**
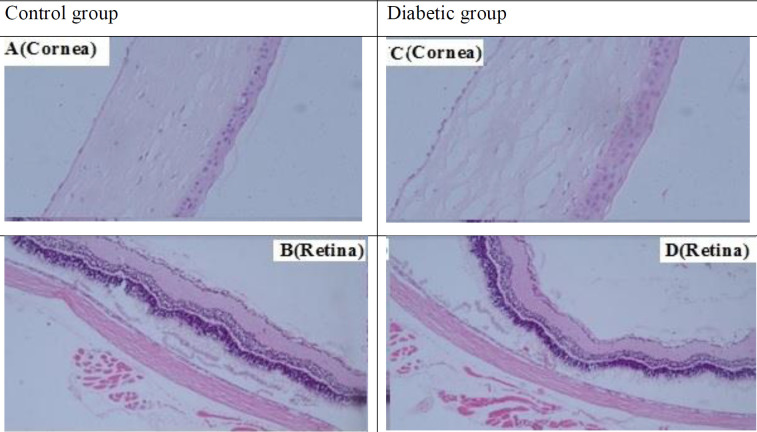
Histological features of the eye tissue in control and type 2 diabetic rats. Tissue sections were stained by hematoxylin-eosin staining and evaluated with the same magnification for all images (40x). Light microscopic examination of ocular sections of rats shows a new natural arrangement in the cornea and retina (Figures 4 A and B). In diabetic rats, however, changes in the structure of the cornea due to breaking cross-links and detachment of the retina from the back of the eye are a good indication of ocular injury (Figures 4 C and D)

Histological features of the eye tissue in control and type 2 diabetic rats. Tissue sections were stained by hematoxylin-eosin staining and evaluated with the same magnification for all images (40x). Light microscopic examination of ocular sections of rats shows a new natural arrangement in the cornea and retina (Figures 4 A and B). Light microscopic examination of ocular sections of diabetic rats showed a change in the structure of the cornea due to the loss of cross-links between the connective fibers in the cornea. In this case, the thickness of the cornea is high but weak (Figures 4A and B). Detachments of the retina from the back of the eye and its thickening are due to BRB breakdown, which often occurs in association with changes in electrolyte homeostasis in Muller cells, a good indication of ocular injury (Figures 4C and D)

## Discussion

The results showed that eating an HFD for 3 months caused hyperlipidemia, hyperglycemia and insulin resistance, inflammation, and oxidative stress in rats, all of which are manifestations of type 2 diabetes. Consistent with these findings, many studies have shown that HFD induces dyslipidemia, hyperglycemia, hepatic steatosis, insulin resistance, oxidative damage, and inflammation in various organs of the body in animal models (23, 24). Similar to previous studies (25-27), hyperglycemia, inflammation, and oxidative stress along with hypertension can be the main pathomechanisms for diabetic retinopathy in this study. Although arterial pressure was not evaluated in diabetic rats, other studies have shown that consuming an HFD can cause high blood pressure(28). Systemic and local hypertension decrease retinal perfusion and induce retinal ischemia/hypoxia which increase free radicals, chemokines, growth factors, and cytokines in the retina, and apoptosis in endothelial cells (29, 30). Under hyperglycemic-hypertensive conditions, the levels of free radicals, cytokines, growth factors, and chemokines increase in the vitreous fluid of diabetic patients and the retina is prone to damage (30, 31). Retinal hypoxia also stimulates the NF-ĸB and HIF-1α signaling pathways and their downstream pathways such as MIP-1α and MCP-1, which attract macrophages and monocytes to hypoxic regions. Macrophages and microglia, along with vascular and retinal immune cells, release TNF-𝛼 and IL-1β, MCP-1, and VEGF into vitreous. These vitreous mediators are involved in the pathogenesis of retinopathy via a direct effect on endothelial cells and indirectly by leukocyte recruitment and activation. Leukocytes in turn produce free radicals, inflammatory cytokines, growth factors, and vascular permeability factors that lead to leukocytosis. Inflammatory cytokines released by leukocytes also activate VEGF and destroy the blood-retinal barrier (BRB) that are clearly seen in the eye tissue sections of rats with type 2 diabetes (31). In fact, in this condition, it causes retinal detachment, as shown in Figure 4D.

Our results showed that the expression of NF-κB and levels of TNF-α and IL-1β significantly increased in eye tissue of diabetic rats, which represents an inflammatory situation in this tissue. NF-κB as a transcription factor is expressed in endothelial cells and retinal pericytes by hypoxia and hyperglycemia (32). NF-κB induces pro-inflammatory cytokine synthesis such as TNF-α and IL-1β and chemokines that they damage to cells through apoptosis and subsequent complications. A condition that occurs well in most tissues of the body in diseases such as diabetes which in addition to dysfunction, also changes the natural structure of cells and tissues. Due to the accumulation of macrophages and monocytes in the ischemic region, it appears that the ocular level of TNF-α and IL-1β is higher than that at the systemic level (33). TNF-𝛼 and IL-1β induce angiogenesis and increase retinal endothelial permeability and induce leukocyte adhesion and oxidation (34). In our study, the MCP-1 level increased in the eye tissue in diabetic rats, which may be due to an increase in NF-ĸB gene expression. MCP-1 is a potent activator and recruiter of macrophages and stimulates the migration of monocytes and macrophages in tissues and induces VEGF production (34, 35). Furthermore, our results showed that the level of MIP-1α significantly increased in the eye tissue of diabetic rats which is consistent with previous studies (36). The association between macrophage activation and retinal angiogenesis has been well demonstrated. It is reported that the presence of macrophages in the hypoxic region locally affects the expression of MCP-1 and is involved in the pathogenesis of diabetic retinopathy (37, 38). MCP-1 levels have been shown to increase in the vitreous of diabetic patients and are higher than in the serum (38). Similar to TNF-𝛼, it was reported that there was a significant relationship between vitreous MCP-1 level and the severity of diabetic retinopathy (38). High levels of MCP-1 are known as retinal inflammation due to diabetes, and its production from endothelial cells, retinal cells, and glial cells are increased with hyperglycemia (39). MIP-1α mediates the synthesis and secretion of TNF-α, IL-1, and IL-6 from fibroblasts and macrophages. Macrophages along with leukocytes produce free radicals, TNF-α, and VEGF which in fact creates a vicious cycle and worsens diabetic retinopathy. 

The eye, especially the retina, is highly perfused and oxygenated due to its high metabolic activity and contains higher concentrations of unsaturated fatty acids than other tissues, so it is prone to damage due to inflammation and oxidative damage by accumulation of macrophages and leukocytes (40). Inflammation and oxidative damage, in addition to changes in cell function, cause changes in their structure and damage tissue, which can be seen in the tissue evaluation of the retina and cornea in ocular tissue samples of diabetic rats in this study. Our results in line with previous evidence (40-42) showed that expression of Nrf2 and HO-1 along with the activity of antioxidant enzymes reduced in the eye tissue of diabetic rats. These results clearly indicated that oxidative damage occurred in ocular tissue. It is possible that free radicals produced by macrophages and leukocytes reduce the activity of antioxidant enzymes and induce oxidative damage in the eye tissue of diabetic rats. The Nrf2/HO-1 pathway has a very prominent role in controlling oxidative damage and has been targeted by many researchers as one of the therapeutic targets in DM and its complications. A study (2017) showed that the Nrf2 signaling pathway via interaction with the NF-κB pathway and regulation of NLRP3 inflammasome has been able to improve animal models of inflammatory diseases (43). Nrf2/HO-1 pathway can regulate angiogenesis through HIF-1α/VEGF pathways and protect the retina from oxidative damage induced by hyperoxia (44, 45). Hence, identifying the interaction of the Nrf2/HO-1 pathway with the signaling pathways of HIF-1α and NF-ĸB plays an important role in controlling diabetic retinopathy and even discovering a drug (11). Two studies (2011, 2015) showed that the miR-146a level significantly decreased in patients with T2DM (46, 47). Alipoor *et al*. (2017) showed in a meta-analysis that level of miR-146a is associated with prevalence of T2DM and its complications(48). Moreover, a study (2014) indicated that a decrease in miR-146a level is associated with the prevalence of type 2 diabetes (49). Now, according to these explanations, the question arises that in these disease conditions, how miR-146a-5p regulates Nrf2 and NF-κB expression and inflammation and oxidative stress status, and what are the responses created following these changes? In answering this question, you should pay attention to several points. The first is that hyperglycemia is a major cause of diabetic retinopathy. It was well shown that miR-146a-5p through MED1 (Mediator of RNA polymerase II transcription subunit 1) plays a significant role in the metabolism of glucose and lipids (50). Although we did not evaluate the correlation between glucose and blood lipids with this microRNA in this study, this association between its levels with HOMA-IR was evaluated. Our results showed that miR-146a-5p had a negative correlation with HOMA-IR (insulin resistance). Therefore, this microRNA can be involved in hyperglycemia and dyslipidemia. It is also well shown that HOMA-IR is significantly associated with oxidative damage and inflammation status (51, 52). Our results also showed that there is a positive correlation between miR-146a with Nrf2 expression and a negative correlation between miR-146a with NF-κB. Nrf2 and NF-κB are both transcription factors that are activated inside the cell in response to their stimuli and then enter the nucleus. Hyperglycemia may activate several signaling adaptor proteins, like protein kinase C (PKC) and Toll-like receptor (TLRs), thus triggering the downstream NF-κB-mediated inflammatory cascade (53). A study (2018) showed that miR-146a is involved in oxidative stress induced by hyperglycemia. They showed that miR-146a/NAPDH oxidase4 pathway inhibits overproduction of ROS, oxidative stress and inflammation, and suppresses the protein expression of VCAM 1 and ICAM 1 in mice with diabetic nephropathy (17). In addition, high glucose could disturb mitochondrial metabolism and aggravate ROS production via triggering the activation of NADPH oxidase. A large amount of ROS can intensify the NF-κB signaling pathway and ultimately exacerbate oxidative stress in diabetics (54, 55). In detail, inflammatory cells could respond to oxidative stress by releasing various NF-κB-mediated pro-inflammatory mediators, which in turn aggravate the status of inflammation and oxidative stress, thereby establishing a vicious cycle (55). Moreover, some studies have shown that miR-146a acts as a regulator of NF-κB and Nrf2 (55-57). MiR-146a could inhibit the activation of NF-κB mediated inflammation via suppressing its target gene expressions like IRAK1 and TRAF6 (58). A study (2017) reported that the level of miRNA-146a is associated with the severity of inflammation in rats (59). A research in 2015 showed that mice lacking the miR-146a gene develop inflammatory disorders and the activity of miR-146a as a molecular brake plays an essential role in inhibiting inflammation (60). Studies have also shown that miR-146a directly reduces inflammatory cytokines production in macrophages (61). Another study showed that miR-146a modulates TLR signaling and cytokine response, and affects the activity of the innate immune system (62). Decrease in miR-125b expression or increase in miR-146a-5p expression by inhibiting NF-κB signaling pathways improves endothelial function and prevents the progression of diabetic complications (63). Moreover, increasing miR-146a-5p inhibits inflammation and oxidative stress (64)>=´W, while decreased miR-146a-5p is associated with inflammation and oxidative stress induced by hyperglycemia (55). The role of miR-146a in NF-κB expression in endothelial cells under hyperglycemic conditions was also evaluated by other researchers (63). In addition, NF-κB could repress the Nrf2- antioxidant responsive elements (ARE) pathway through interaction with Keap1 (65). MicroRNA146a/NAPDH oxidase4 also decreases ROS generation and inflammation in a diabetic nephropathy model (17). Nrf2 associates with small Maf proteins (sMaf) and binds to ARE in the promoters of its target genes. This process is essential for the assembly of the RNA polymerase machinery and initiation of transcription (66). Nrf2 can either be degraded in the nucleus via the β-TrCP–GSK3β axis or alternatively, it may translocate back to the cytoplasm where it is swiftly degraded by Keap1 (67). Intracellular levels of Nrf2 are highly regulated by oxidizing (ROS) and electrophilic agents such as plant phenolic compounds. Hence, changing the cellular levels of antioxidants as a therapeutic strategy can have a significant impact on improving this condition. Thus, a low level of miR-146a could be involved in insulin resistance and diabetic complications, due to changes in the expression of genes involved in inflammation, oxidative stress, and glucose and lipid metabolism. On the other hand, miR-146a along with miR-155, miR-125b, and miR-150 are involved in the complications of diabetes (12). A similar study (2016) to investigate the role of miR-150 in HFD-induced diabetic retinopathy, showed that overexpression of miR-150 in endothelial cells reduces the expression of vascular endothelial factor 2 receptor protein (VEGFR2). They concluded that miR-150 plays an important role in the pathogenesis of retinal angiogenesis in HFD-induced type 2 diabetic rats, which is mediated by VEGFR2 (12). Recent findings suggested that circulating miRNAs can make effective connections between different cells and organs, they are involved in the activation or inactivation of target gene transcription by binding to promoter regions and in post-transcriptional regulation of gene expression (3). 

**Table 1:** The primer sequences used in this study

Gene	Sequence	Product length (bp)
Nrf2	F 5´- AAAGACAAACATTCAAGCCGATTAG -3´ R 5´- TTGCTCCTTGGACATCATTTCAT -3´	141
HO-1	F 5′- TTAAGCTGGTGATGGCCTCC -3′ R 5′- GTGGGGCATAGACTGGGTTC -3′	90
NF-κB	F 5′- GCACCAAGACCGAAGCAAT-3′ R 5′- CGTAACCGCGTAGTCGAAGA -3′	143
TNF-α	F 5′- GGTCCCAACAAGGAGGAGAAGT -3′ R 5′- GGTGGTTTGCTACGACGTGG -3′	130
β-actin	F 5′- CCCAGGCATTGCTGACAGG -3′ R 5´- TGGAAGGTGGACAGTGAGGC-3′	141

*: *P*<0.05

* and ** indicate *P*<0.05 and *P*<0.01, respectively

**Table 2:** Mean ± SD of serum levels of glucose, insulin, and HOMA-IR in basal and final levels in control and diabetic groups

**Factor** **Group**	**SOD(U/mgprotein)**	**CAT(U/mgprotein)**	**GPx(U/mgprotein)**	**MDA (mmol/mg protein)**
**Control**	12.26 ± 1.74	18.16 ± 2.54	23.31 ± 1.74	3.16 ± 0.54
**T2DM**	6.34 ± 1.34*	10.10 ± 3.64*	12.24 ± 2.34*	9.36 ± 1.26**

Mismatched lowercase letters indicate a significant difference over time and dissimilar uppercase letters indicate differences between groups. **: *P*<0.01

**Table 3:** Mean ± SD of oxidative biomarker levels in the ocular tissue of control and diabetic groups

**Group/Factor**	**TNF-α (pg/mg)**	**IL-1β (pg/mg)**	**MIP-1α (pg/mg)**	**MCP-1 (pg/mg)**
**Control**	8.26 ± 0.74	3.782 ± 1.14	7.02 ± 0.13	3.23 ± 0.18
**T2DM**	19.14 ± 1.04*	8.21 ± 1.26*	16.1 ± 0.96*	13.18 ± 0.36**

* and ** indicate *P*<0.05 and *P*<0.01, respectively

**Table 4:** Mean ± SD of the levels of proinflammatory cytokines, MCP-1, and MIP-1α in the ocular tissue of control and diabetic groups of rats

**Group/Factor**	TNF-α (pg/mg)	IL-1β (pg/mg)	MIP-1α (pg/mg)	MCP-1 (pg/mg)
control	8.26 ± 0.74	3.782 ± 1.14	7.02 ± 0.13	3.23 ± 0.18
T2DM	19.14 ± 1.04^*^	8.21 ± 1.26 ^*^	16.1 ± 0.96 ^*^	13.18 ± 0.36 ^*^

* and ** indicate *P*<0.05 and *P*<0.01, respectively

* and ** indicate *P*<0.05 and *P*<0.01, respectively

## Conclusion

It can be concluded that a decrease in miR-146a could be involved in diabetic retinopathy and insulin resistance, possibly due to changes in the expression of genes involved in inflammation, oxidative stress, and glucose and lipid metabolism. It is also suggested that changes in serum or tissue levels of miR-146a may be an important indicator in predicting diabetic complications. In addition, given its widespread role, it can be a potential therapeutic agent.

## Authors’ Contributions

The work presented in this article was carried out through collaboration between all authors. SAR and AA made the initial hypothesis. All authors participated in defining the research theme and providing the proposal. AA performed the experiments on animal models. KHN performed the molecular measurements. AA, KHN, and SAR interpreted the data, wrote and edited the article. AA supervised the work. All authors have contributed to, edited, and approved the article.
